# Platelet Rich Plasma Versus Autologous Conditioned Serum in Osteoarthritis of the Knee: Clinical Results of a Five-Year Retrospective Study

**DOI:** 10.7759/cureus.24500

**Published:** 2022-04-26

**Authors:** Hüseyin Sina Coşkun, Alparslan Yurtbay, Ferhat Say

**Affiliations:** 1 Orthopaedics and Traumatology, Ondokuz Mayıs University, Samsun, TUR; 2 Orthopaedics and Traumatology, Samsun Education and Research Hospital, Samsun, TUR

**Keywords:** serum, autologous, platelet-rich plasma, knee, osteoarthritis

## Abstract

Objectives: There is no consensus on the effectiveness of platelet-rich plasma (PRP) and autologous conditioned serum (ACS) in the treatment of knee osteoarthritis (OA). Also, the group of patients who will benefit most from this treatment is not clear. This study aims to understand the effects of two treatment modalities: ACS and PRP on pain and clinical scores in the treatment of OA. For this reason, we compared the long-term (five-year follow up) clinical results of the patients to whom these two treatment methods were applied.

Materials and methods: Eighty-two knee osteoarthritis cases, selected from a database prospectively maintained in our tertiary university hospital after institutional ethics committee approval, examined between January 2013 and September 2020 and treated with ACS and PRP by the same orthobiological treatment team, were retrospectively analyzed. The clinical results of group A (n=40) treated with ACS and group B (n=42) treated with PRP were statistically analyzed. Clinical evaluations were made pre-injection and at one, six, 12, 24 and 60 months post-treatment, using the knee injury and osteoarthritis result score (KOOS) for the evaluation of function and a visual analog scale (VAS) for the evaluation of pain.

Results: Side effects were noted in two patients (5%) in group A and 16 patients (38.1%) in group B. More side effects were seen in group B compared to group A (p<0.001). The better VAS scores in both groups were detected in the sixth and 12th months. When VAS scores were examined, better results were obtained in group A in the 12th and 24th months (p<0.05). When KOOS scores were examined, the superiority of ACS to PRP at 12 and 24 months was shown in KOOS.S, KOOS.P and KOOS.ADL scores (p<0.05). There was no statistically significant difference between the two groups in terms of all scores and baseline scores at 60 months.

Conclusion: The effectiveness of ACS and PRP treatments can last up to two years. After two years, the effectiveness of both treatments decreases. Comparing the two treatments, ACS treatment showed better results on VAS and KOOS scores compared to PRP treatment.

## Introduction

Osteoarthritis (OA) is a heterogeneous, progressive, complex joint disease that develops as a result of impaired joint cartilage integrity, leads to clinical and radiological findings, and causes changes in bone and joint [1]. Osteoarthritis is the most common joint disease in the world. It is the most common cause of pain and loss of function in adults in Western societies [2]. Currently, there are no definitive treatment methods for OA. Therefore, many drugs and treatment options are being studied for use in OA treatment. The ideal treatment should stop or slow the progression of OA and have little or no side effects.

For many years, the pathogenesis of OA is based on the thesis that cartilage degeneration develops as a result of prolonged mechanical load on the joint. Nowadays, it is known that the pathophysiology of the disease, what products formed in anabolic and catabolic processes play a role and is a disease that concerns the entire joint [3]. In the pathogenesis of OA, several treatment methods are being developed to prevent or slow the progression of the disease by targeting the inflammatory process in which cytokines, chemokines, growth factors, matrix metalloproteinases, synoviocytes and chondrocytes play a role. These drugs are called disease-modifying osteoarthritic drugs (DMOADs). Cytokines and growth factors are now known to play an important role in the pathogenesis of OA [3]. Interleukin-1 (IL-1) is one such pro-inflammatory cytokine, suspected to play a prominent role in the pathophysiology of OA [4]. It stimulates matrix metalloproteinases and prostaglandin production, both of which negatively impact the cartilage matrix integrity [5]. Disease-modifying osteoarthritic drugs are believed to intervene with the inflammatory pathways of these cytokines thereby slowing down disease progression, decreasing disease symptoms, and improving quality of life [6].

Autologous conditioned serum (ACS) is a serum isolated from whole blood, incubated and then separated with centrifugation. There are IL-1 receptor antagonists (IL-1ra) and anti-inflammatory cytokines such as IL-4, IL-10, and IL-13 in this serum. Thus, ACS has the potential to become DMOADs. The potentially beneficial effect of ACS therapy on the symptoms and progression of OA disease has been demonstrated in several randomized controlled clinical trials [7-9].

Platelet-rich plasma (PRP) is a liquid that contains many growth factors. These factors stimulate cell proliferation, cellular migration, angiogenesis and extracellular matrix production [10]. The high number of platelets in the PRP accelerates the natural healing process when too much growth factor is applied to the target area where the damage occurs. Thus, PRP has the potential to become DMOADs.

In the OA treatment guide published by Osteoarthritis Research Society International (OARSI), pharmacological and non-pharmacological treatment options are recommended [11]. Platelet-rich plasma and ACS are not included in the guideline published by OARSI. However, in many countries, these treatment methods are discussed and used by physicians and patients in the treatment of OA. Both PRP and ACS are the treatment methods that attract the attention of physicians and patients. For this reason, many studies on PRP and ACS treatment methods have been published and continue to be published [12-19]. This study aims to understand the effects of the two new and popular treatment methods i.e., ACS and PRP, on pain and clinical scores in OA treatment.

## Materials and methods

This study was designed as a retrospective comparative clinical study (Level 3). Between January 2013 and September 2020, 3756 patients were admitted to our clinic with complaints of knee pain and diagnosed with OA. Out of which, 82 patients were eligible to participate in the study according to the inclusion and exclusion criteria identified. Patients in group A (n=40) were given ACS injections and group B (n=42) were given PRP injections.

Statistical analysis was applied to a total of 82 patients (group A, n=40; group B, n=42). Group A was treated with ACS and group B with PRP and was statistically analyzed. Clinical evaluations were made pre-injection and at one, six, 12, 24 and 60 months post-treatment, using KOOS for the evaluation of function and VAS for the evaluation of pain. The mean follow-up period in both groups was 60 months. The study was performed at the Department of Orthopedics and Traumatology at Ondokuz Mayıs University Faculty of Medicine in Samsun and was approved by the local ethical committee of the Ondokuz Mayıs University (approval no. OMÜ KAEK 2020/759).

Patient selection

The diagnosis of OA was made according to the American College of Rheumatology criteria [1]. Inclusion and exclusion criteria were determined before starting the study. Inclusion criteria: patients over the age of 18 and admitted to the clinic with OA symptoms (pain, stiffness, disability), stage 2-3 primary OA patients according to Kellgren/Lawrence (K/L) staging [20] were included voluntarily. Exclusion criteria: patients who have previously received injection therapy in the same knee and are over 80 years old; patients diagnosed with coxarthrosis, neurovascular disease, any infectious disease, coagulopathy, thrombocytopenia, anemia, known immunodeficiency disease, osteomyelitis; crystalline, inflammatory and infectious arthropathies; corticosteroid and anti-coagulant usage or morbid obesity; bilateral symptomatic lesions, secondary OA, stage 1-4 OA patients according to K/L staging. After the patients were informed about the study, their written and verbal consent was obtained and included in the study. Bilateral knee injection was not applied.

ACS preparation

First, 50 ml of venous blood was collected from all patients who participated in the study. The orthokine syringe containing chromous sulfate (CrSO4) coated glass beads were used in the blood collection procedure. The syringe was gently rotated for full contact of blood with glass-coated beads inside the syringe. The blood taken was incubated at 37^o^ for 12 hours. In this way, the production of IL-1ra by white blood cells was induced [7,8]. After the incubation period, centrifugation was carried out and then the serum supernatant portion was separated. At the end of these processes, four 3 ml injections were obtained. These injections prepared were stored at -20 degrees for later use.

PRP preparation

First, 32 ml of venous blood was collected from patients. The blood was collected in 4.5 ml, eight sterile tubes with 3.2% sodium citrate as an anticoagulant. An average of 4 ml of blood was collected in each tube. The tubes were then spun for 10 minutes at 1800 rpm on a table-top centrifuge once. A total of 12 ml of PRP was collected, 1.5 ml from each tube. At the end of these processes, four 3 ml injections were obtained. These injections prepared were stored at -20 degrees for later use. For platelet activation, 5.5% calcium chloride (CaCl2) (50 µl CaCl2 in 1 ml PRP) was used. The product is type 2A as per the Mishra classification [21]. In our PRP preparation and implementation procedure, the PRP was developed by making use of the methods used in the works of Anitua [22].

Follow-up and outcome measures

Patients in both groups received three consecutive injections (3 ml per injection), one week apart. Patients were injected in the supine position, through the knee superolateral portal. Sterility was taken into consideration during blood collection, centrifugation, separation, storage and application processes. Follow-up visits were done at one, six, 12, 24 and 60 months from the initial intra-articular injection.

The VAS and KOOS scores of the patients were noted at each interview. Each interview was made by the same orthopedic physician. The VAS is usually 10 cm long, in the form of a connected horizontal line, and both ends represent extreme values. We asked the patients to think about the pain they felt in the last 24 hours and to mark a point on this line. This marked point represents the pain value that patients have been feeling for the last 24 hours. Pain is given out of 10 points in this chart. The test has proven itself for a very long time and is accepted in world literature, and is safe and easy to apply [23].

Knee injury and osteoarthritis outcome score is a valid, reliable and responsive self-scoring system that can be used for short-term and long-term monitoring of various knee injuries, including osteoarthritis. The most important advantage of this scoring system is the evaluation of many knee injuries that can cause OA and long-term OA, as well as its use in both short and long-term follow-up. The KOOS collects data on five knee-specific patient-centered subscales: (1) pain (KOOS.P); (2) other symptoms such as swelling, stiffness, limited range of motion, and mechanical symptoms (KOOS.S); (3) disability at the level of daily activities (KOOS.ADL); (4) disability at a physically more challenging level than daily life and sports activities (KOOS.SP); (5) mental and social aspects such as awareness and lifestyle changes (KOOS.QL). The Likert scale is used to answer each question, and all items have 0 (no problem) and 4 (very severe) and five possible answer options for each of the five. Scores are converted to a scale of 0-100, of which zero represents over-knee problems, and 100 does not commonly represent knee problems in orthopedic evaluation scales and generic measurements. It is suggested that a minimum change of eight to 10 points or above in the KOOS score should be considered significant [24]. It has been reported that KOOS is more effective than The Western Ontario and McMaster Universities Arthritis Index (WOMAC) score in young patients with a knee injury [25].

Statistical analysis

The data obtained were evaluated with Statistical Package for Social Sciences (SPSS) version 20.0 (IBM corp., Armonk, NY, USA). Results are given as mean ± standard deviation for normally distributed data and median (minimum, maximum) for non-normally distributed data. When comparing the two independent groups, if the data were normally distributed, the two independent t-tests were used. If there was no normal distribution, the Mann Whitney U test was used. The values ​​of each group at different times of three or more were evaluated by repeated measurement analysis. Repetitive measurement analysis and Friedman test were used. P-values less than 0.05 were considered statistically significant.

This study was conducted following the principles of the Declaration of Helsinki and with the patients’ permission expressed through written consent.

## Results

Demographics and baseline characteristics

The study included 82 patients who were split into two groups. Both groups were followed retrospectively for an average of 60 months. Statistical analysis was performed on a total of 82 patients. There were no significant differences between the two groups according to sex, body mass index (BMI), K/L OA grade, baseline scores of VAS and KOOS subscales (p>0.05). There were significant differences between the two groups according to age, range of motion (ROM), and follow-up time (p<0.05). Baseline characteristics of the two groups are shown in Table 1.

**Table 1:** Baseline characteristics of the two groups PRP: Platelet-rich plasma, M: Male, F: Female; BMI: Body mass index, K/L: Kellgren/Lawrence classification, VAS: Visual analog scale, KOOS: Knee injury and osteoarthritis outcome score, ADL: Activity in daily living, Sport/Rec: Function in sport and recreation, ROM: Range of motion.  * p<0.05 **p<0.001

	Group A (n=40)	Group B (n=42)		P -value (Between Groups)
Age, mean ± SD (range), years	56.68 ± 8.96		50.79 ± 10.67	0.008*
Sex, M: F, n	28:12		32:10	
BMI, mean ± SD (range)	30.04 ± 5.30		30.05 ± 4.82	0.626
K/L grade, n (%)				
2	27 (67.5)		30 (71.4)	
3	13 (32.5)		12 (28.6)	
VAS score, mean ± SD	6.93 ± 1.40		7.07 ± 1.71	0.523
KOOS score, mean ± SD				
Symptom	58.57 ± 18.9		64.96 ± 16.8	0.099
Pain	40.90 ± 14.4		48.88 ± 18.2	0.058
ADL	43.27 ± 13.32		51.93 ± 19.1	0.053
Sport/Rec.	25.50 ± 15.76		32.98 ± 22.7	0.203
QOL	30.31 ± 15.21		36.31 ± 18.3	0.121
ROM, degree	120.6 ± 9.5		121.3 ± 9.8	0.000^**^

Side effects observed in patients during the five-year follow-up period were noted. The distribution of the side effects seen by groups is shown in Table 2. All the side effects were seen within the first month after injection. Side effects were noted in two patients (5%) receiving ACS therapy and 16 patients (38.1%) receiving PRP therapy. More side effects were seen in the patient group treated with PRP compared to the patient group treated with ACS. This situation was found to be statistically significant (p<0.001).

**Table 2:** Distribution of side effects by groups

	Patients with side effects n (%)	Patients with no side effects n (%)
Group A (n=40)	2 (5)	38 (95)
Group B (n=42)	16 (38.1)	26 (61.9)

VAS pain scores

**Figure 1:**
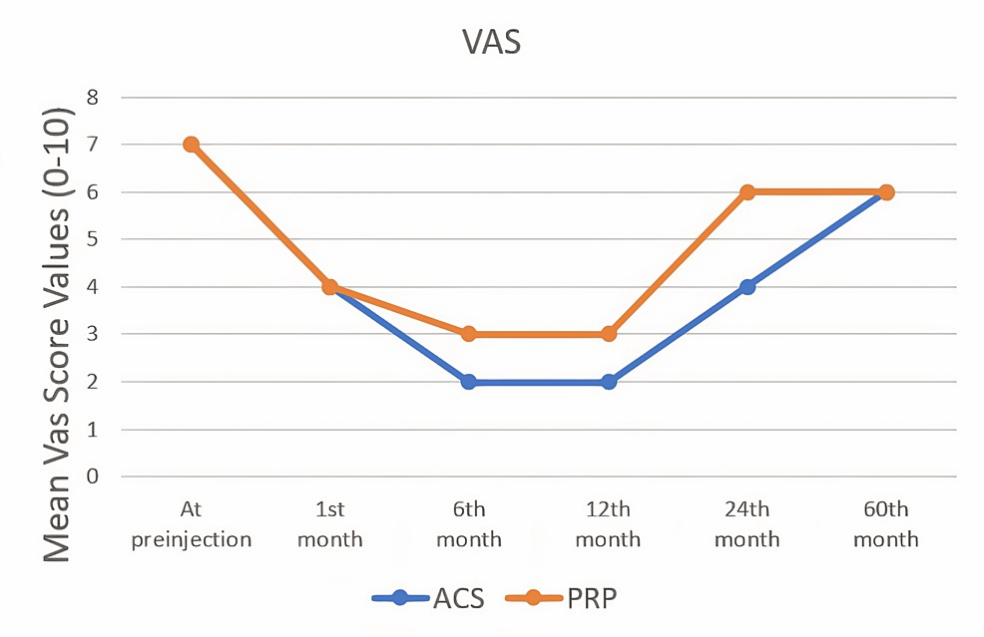
The changes of VAS scores by months

The VAS scores of all patients were noted before the injection and at one, six, 12, 24 and 60 months. The statistical results of the VAS score values ​​of both groups and the comparison of these values ​​are shown in Table 3. The better VAS score values ​​were reached at the end of the 12th month in both treatment groups. A statistically significant difference was found in the VAS scores between the two treatment groups at the 12th and 24th months (p<0.05). Although both treatment groups decreased the VAS score values, it was observed that the ACS group decreased the VAS scores more at 12th and 24th months (Figure 1). There was no statistically significant difference between the groups in the VAS score values ​​at the end of the 60 months.

**Table 3:** Comparison of VAS score differences by months * p<0.05 **p<0.001

	Group A (n=40) Median (Min-Max)	Group B (n=42) Median (Min-Max)	P -value (Between Groups)
At pre-injection	7 (4-9)	7 (4-10)	0.523
1^st^ month	4 (2-7)	4 (1-8)	0.731
6^th^ month	2 (0-7)	3 (1-8)	0.760
12^th^ month	2 (0-7)	3 (0-8)	0.022*
24^th^ month	4 (1-8)	6 (3-10)	0.00**
60^th^ month	6 (2-9)	6 (3-10)	0.144

Clinical outcomes

KOOS Scoring System

**Figure 2:**
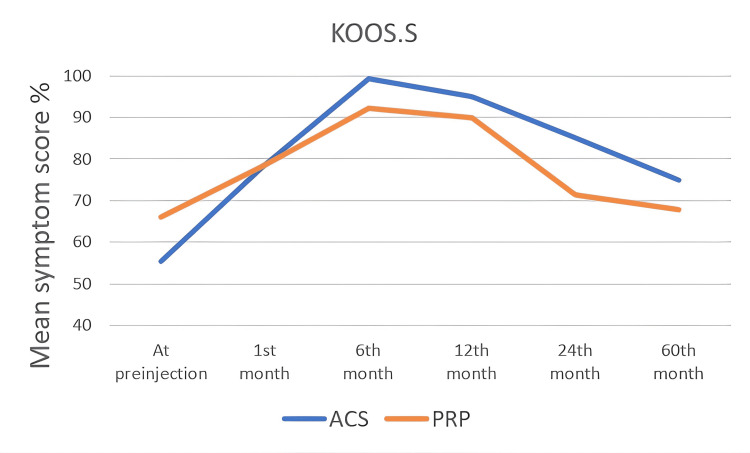
The changes in KOOS.S scores by months

**Figure 3:**
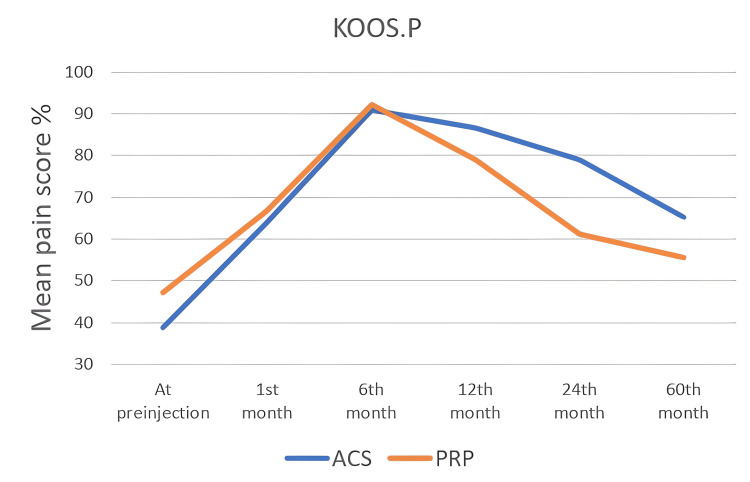
The changes in KOOS.P scores by months

**Figure 4:**
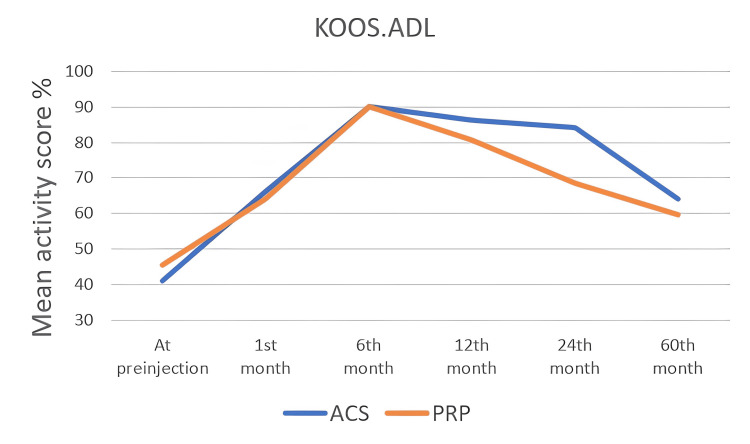
The changes in KOOS.ADL scores by months

**Figure 5:**
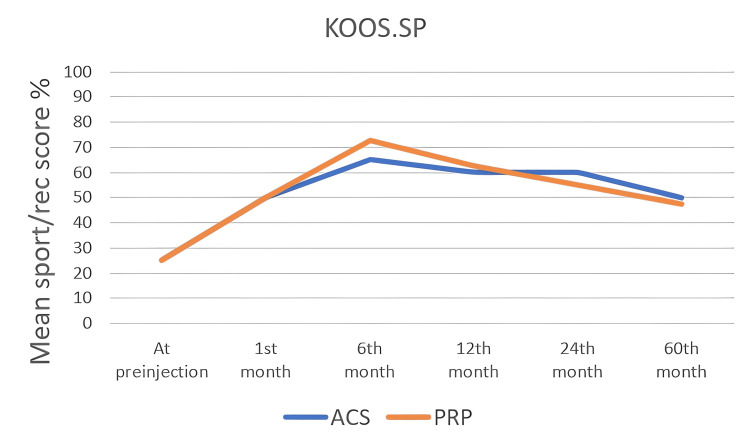
The changes inf KOOS.SP scores by months

**Figure 6:**
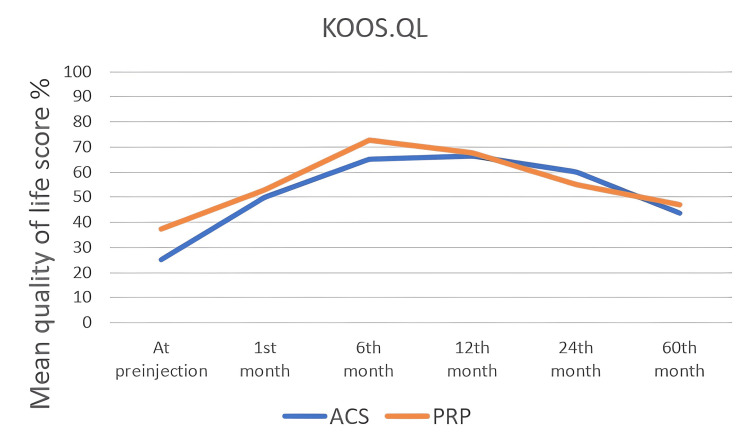
The changes in KOOS.QL scores by months

The KOOS score values ​​of all patients were noted in the first interview and at one, six, 12, 24, and 60 months. In the KOOS scoring system, which consists of five subtitles, each subtitle was noted and analyzed statistically, separately. In all KOOS score subtitles, score changes within both groups were found to be statistically significant (p<0.05). When the groups were compared with each other, a statistically significant difference was found at the 12th and 24th months (Table 4) and (Figure 2, 3, 4, 5, 6). There was no statistically significant difference between the groups in the KOOS score values ​​at the end of the 60 months. When both groups were compared, the superiority of ACS to PRP at 12 and 24 months was shown in KOOS.S, KOOS.P and KOOS.ADL scores.

**Table 4:** Comparison of KOOS scores changes by months * p<0.05 **p<0.001

	Group A (n=40) Median (Min-Max)	Group B (n=42) Median (Min-Max)	P -value (Between Groups)
KOOS.S			
At pre-injection	55.36 (25 - 93)	66.08 (32 – 100)	0.099
1^st^ month	78.57 (32 – 100)	78.57 (25 – 100)	0.896
6^th^ month	99.29 (56 – 100)	92.14 (53 – 100)	0.141
12^th^ month	95 (49 – 100)	90 (53 – 100)	0.082
24^th^ month 60^th^ month	85 (39 – 100) 75 (25 – 100)	71.43 (43 – 100) 67.86 (36 – 100)	0.018* 0.449
KOOS.P			
At pre-injection	38,9 (16,7 - 72,2)	47,2 (22,2 - 86,1)	0.058
1^st^ month	63,9 (36,1 - 83,3)	66,7 (27,8 - 97,2)	0.985
6^th^ month	90,8 (40 - 100)	92,2 (39,4 - 100)	0.757
12^th^ month	86,7 (40,6 - 108,9)	78,9 (40,6 - 110)	0.014*
24^th^ month	78,9 (25 - 115)	61,1 (30,6 - 98,3)	0.002*
60^th^ month	65,3 (22,2 - 100)	55,6 (30,6 - 97,2)	0.379
KOOS.ADL			
At pre-injection	41,2 (22,1 - 77,9)	45,6 (25 - 89,7)	0.053
1^st^ month	66,2 (32,4 - 88,2)	64 (29,4 - 100)	0.692
6^th^ month	90 (40 - 109,1)	90 (45,9 - 109,1)	0.329
12^th^ month	86,3 (33,5 - 112,1)	80,6 (40,9 - 112,1)	0.049*
24^th^ month	84,1 (23,5 - 115)	68,4 (30,9 - 106,2)	0.000**
60^th^ month	64 (22,1 - 100)	59,6 (23,5 - 92,7)	0.565
KOOS.SP			
At pre-injection	25 (5 - 55)	25 (0 - 80)	0.203
1^st^ month	50 (10 - 75)	50 (0 - 95)	0.660
6^th^ month	65 (10 - 105)	72,5 (10 - 100)	0.989
12^th^ month	60 (5 - 100)	62,5 (5 - 100)	0.475
24^th^ month	60 (0 - 100)	55 (0 - 95)	0.154
60^th^ month	50 (5 - 100)	47,5 (0 - 95)	0.657
KOOS.QL			
At pre-injection	25 (0 - 68,8)	37,5 (0 - 75)	0.121
1^st^ month	50 (25 - 75)	53,1 (0 - 100)	0.925
6^th^ month	65 (27,5 - 91,3)	72,5 (10 - 100)	0.639
12^th^ month	66,3 (30 - 86,3)	67,5 (5 - 100)	0.738
24^th^ month	60 (22,5 - 100)	55 (0 - 93,8)	0.068
60^th^ month	43,8 (12,5 - 100)	46,9 (0 - 93,8)	0.678

## Discussion

Osteoarthritis involves the entire synovial joint and is a complex disease with no definitive curative treatment. Although its etiology is not fully understood, some risk factors such as previous injury or gender are known to increase the risk of developing knee OA [26,27]. Today, many treatment methods are developed for OA disease. The described pharmacological and non-pharmacological treatment methods for this disease have advantages and disadvantages in themselves. These treatment modalities provide advice to clinicians with well thought out and well-written treatment guidelines [11,28]. Although these treatments are rarely included in international treatment guidelines, doctors and patients discuss and use them in the treatment of OA in many countries.

In recent years, studies have been published that provide a better understanding of the factors that cause OA. New treatments are being developed for joint cartilage defects. As the agents that cause OA development are better understood, it becomes easier to develop drugs that target these agents and slow down the development of OA. These drugs are called DMOADs. The IL-1 plays an important role in chronic degeneration of the knee joint due to its pro-inflammatory effect [3-5]. As a result, IL-1 receptor antagonist appears to be an important agent in slowing the development of OA.

 Autologous conditioned serum therapy is very interesting because it is obtained from the patient's own blood compared to anakinra that is a synthetic IL-1 receptor antagonist (IL-1 Ra). Treatment with ACS is becoming important in terms of reducing drug side effects and costs. It has been reported in studies that ACS therapy is successful in the treatment of knee OA [7,8,17-19].

Baltzer et al., in a prospective, double-blind clinical study examining the effects of ACS treatment on knee OA, demonstrated that ACS is a safe and effective treatment and its effect continues for up to two years. In this study, patients received a total of six knee injections at three-week intervals. The VAS and WOMAC scores of the patients were noted at seven, 13, 26 and 104 weeks. The ACS treatment has been shown to have a significant positive effect on scores compared to hyaluronic acid (HA) and placebo [7]. In our study, the most favorable change in pain and clinical scores of patients treated with ACS was seen in the sixth month and the effectiveness of ACS treatment continued for up to two years, similarly.

In another prospective, placebo-controlled clinical study, Yang et al. showed that ACS treatment had statistically significant improvements in VAS and KOOS clinical scores compared to placebo [8]. One-year results of patients in this study are reported. Unlike our study, a total of six intra-articular injections were applied to the patients. New studies are needed to understand the effectiveness of the number of doses on treatment.

A prospective cohort study was conducted to investigate the long-term effect of intra-articular ACS injection. The results of this study showed that ACS injection did not significantly delay the need for knee arthroplasty at a 10-year follow-up compared to placebo [16]. However, in this study, only the incidence of knee arthroplasty was examined in patients. Improvement in symptoms and functions between the first injection and surgery was not evaluated.

The results we obtained with our study are consistent with the medical literature. In this study, randomization and blinding could not be done in the patient selection. Even so, we clearly demonstrated that ACS and PRP have a positive effect on knee joint functions and pain scores. The fact that the patient follow-up period was five years in the study is the strength of the study.

The improvement in VAS and KOOS scores in the first six months to baseline values ​​in both groups was statistically significant (p<0.05). The maximum improvement in scores in both groups was seen at six months. The effectiveness of the treatments continued in both groups until the 24th month. When the scores ​​of the 24th month were examined, the superiority in both VAS and KOOS scores of the ACS treatment group compared to the PRP treatment group was found to be statistically significant (p<0.05). The ACS fluid contains a large amount of IL-1Ra as well as a large number of other cytokines and growth factors. The incubation process stimulates the production of IL-1 Ra along with other cytokines and growth factors [29]. This explains the effect of ACS treatment similar to and above PRP treatment.

The KOOS is a valid, reliable and sensitive self-administered scoring system that can be used for short-term and long-term follow-up of various knee injuries, including OA. The most important advantage of this scoring system is the evaluation of KOOS scoring, OA and many knee injuries that can cause OA in the long term, as well as its use in both short and long term follow-ups [24]. It has greater sensitivity compared to other more general scoring such as WOMAC and the 36-item short-form health survey (SF-36) [24]. It has been reported that KOOS is more effective than WOMAC scoring in young patients with a knee injury [25]. Therefore, we preferred KOOS scoring in our study.

The PRP injection therapy applied to the knee joints of patients with early-stage primary OA seems to be an effective treatment method for pain control and improvement of function in treated patients, as reported in various studies [12-15]. On the other hand, alternative treatment options for patients who are resistant to PRP injection are limited. The effectiveness of products based on autologous blood components in pain and inflammation control is based on the presence of anti-inflammatory cytokines and pro-regenerative factors released by autologous blood cells administered via intra-articular injections [30]. The inability to transfer inflammation-suppressing factors from intracellular compartments to plasma may explain the failure of autologous blood-based therapies. In local treatments derived from autologous blood components, innovative treatment methods can be developed that aim to enhance factor release through ex vivo stimulation of patients' blood cells. Applying this approach produces a patient serum reported to be rich in anti-inflammatory factors, particularly in IL-1 Ra. This may explain why local injection of this form of ACS is superior to PRP therapy for potent anti-inflammatory activity leading to pain control in early-stage forms of OA.

This study has some limitations. All patients included in the study received only one of these two treatments throughout the disease course. The five-year follow-up period is a long time. During this time, we had patients who received another treatment. These patients were excluded from the study. This limitation is one of the most important reasons for the low number of our patients. The study should be designed as a prospective, larger patient population, randomization and placebo-controlled. Thus, the study would be of higher quality in terms of evidence-based scientific medicine. Specifically, cytokines and growth factors were not studied in the fluids obtained from both ACS and PRP treatments. A biological analysis of the joint was not done. Changes that occur with intra-articular biological analysis after injections into the joint can be objectively demonstrated. Also, the number of the dose was determined by the authors as three times for each therapy since there is no consensus in the current literature.

## Conclusions

When the efficacy and superiority of long-term ACS and PRP treatments were examined, it was seen that both treatments were effective in the first two years, but lost their effects after two years, and ACS treatment was more effective than PRP treatment in the first two years. This study demonstrates and confirms that ACS is a safe and effective treatment for knee OA. It can be used as an alternative treatment method, especially when standard OA treatments fail.

Growth factors, cytokines and interleukines can play a key role in stopping or slowing the progression of early-stage OA. Clinical studies on growth factors, cytokines and interleukines in the treatment of OA may provide promising results. There is a need for further randomized, double-blinded, prospective clinical studies with a larger patient population, including all stages of OA of the joint and longer follow-up to clarify these findings.
